# Editorial: Waken the Silent Majority: Principles and Pathogenic Significance of Non-Acetyl Acylation and Other Understudied Post-Translational Modifications

**DOI:** 10.3389/fcell.2022.896324

**Published:** 2022-04-13

**Authors:** Fangliang Zhang

**Affiliations:** ^1^ Department of Molecular and Cellular Pharmacology, University of Miami Miller School of Medicine, Miami, FL, United States; ^2^ Sylvester Comprehensive Cancer Center, University of Miami Health System, Miami, FL, United States

**Keywords:** posttranslational modification, arginylation, arginyltransferase, Ate1, terminal modification, evolution

Arginylation, the ribosome-independent transfer of arginine to protein/peptide, was discovered nearly six decades ago (Kaji et al., 1963; Kaji, 1968). Despite the long history, this posttranslational modification (PTM) remains underexplored. This is hindered by the fact that the study of arginylation is currently only conducted by a few dozen of research groups with a limited number of relevant publications each year.

In a certain sense, the lack of understanding of arginylation even leads to a false impression that this phenomenon may be unimportant. However, arginylation is an exceptionally widespread process that has been found in all eukaryotes examined (Graciet et al., 2006; Graciet et al., 2009; Licausi et al., 2011; Van and Smith, 2020). Such a conservation would be unimaginable if this PTM did does not play some important physiological role.

In this Research Topic “*Waken the Silent Majority: Principles and Pathogenic Significance of Non-Acetyl Acylation and other Understudied Post-Translational Modifications*,” several papers directly relevant to the intriguing phenomenon of arginylation are included to showcase the diverse functions of this PTM and the evolutionary root of arginyltransferase1 (ATE1) (Balzi et al., 1990), the main enzyme catalyzing arginylation in most eukaryotes (Kato and Nozawa, 1984).

In the review paper “Post-translational Modifications of the Protein Termini” by Chen and Kashina, a concise and balanced summary of many different types of PTMs on both the N- and C-termini of proteins are presented for their biochemical mechanisms and potential physiological roles. These include arginylation, which is mostly an N-terminal modification. In the original research paper “Arginylation regulates G-protein signaling present in the retina” by Fina et al., the researchers found that several components in the G-protein signaling complexes in retina are subjected to arginylation, which may in turn affect the retinal function. In another research paper “Protein Posttranslational Signatures Identified in COVID-19 Patient Plasma” by Vedula et al., researchers used comprehensive proteomic approaches and identified significant alterations of arginylation (and several other PTM) signatures in the plasma of COVID-19 patients. While the exact physiological meaning of these changes still awaits further clarification, this finding nevertheless highlights the potential involvement of arginylation in virus-induced pathological conditions. Finally, new clues for interpreting the role of the arginylation enzyme ATE1 was provided in the original research “Regulation of Mitochondrial Respiratory Chain Complex Levels, Organization, and Function by Arginyltransferase 1” by Jiang et al. (contributed by my own research group). In this study, a small fraction of ATE1 was found located inside mitochondria and is essential for the proper function of mitochondria in respiration. Intriguingly, homologues of eukaryotic ATE1 can be traced back to alpha-proteobacteria, relatives of the ancient ancestor of mitochondria. This connection between ATE1 and mitochondria may constitute a new angle for understanding the diverse functions of ATE1 in cellular metabolism (Brower and Varshavsky, 2009; Zhang et al., 2015), stress response (Wiley et al., 2020; Kumar et al., 2016; Deka et al., 2016), and oxygen sensing (Moorthy et al., 2022).

Overall, while arginylation is still a poorly understood process, the papers presented in this Research Topic will help to shorten the gap. Hopefully with more research and further advancements of techniques including proteomic study tools, a more comprehensive picture of arginylation will soon be on the horizon.
